# The Training of the Health Visitor

**Published:** 1915-08-14

**Authors:** 


### THE TRAINING OF THE HEALTH VISITOR.

The health visitor is becoming a much more important person than she was a few years ago, if only because the interests of which she was the representative, and, within obvious limits, the pioneer, have now risen to the status of institutional organisations. A by-product of this, which is significant, is the new nomenclature which is coming into vogue, and, as a necessary result of this, the new class of books which are being written on the subject. For examples of the new institutions which are growing up we have only to mention the mothercraft and child welfare centres which are now springing up, and to which, as we note on another page, at least two Government Departments are now awarding grants. The day nursery, the crèche, the centre for infant consultations, and what is termed antenatal work, with the giving of lectures to mothers, are all new types of institutions, organised and administered with varying degrees of success and repute, and now at the interesting stage of coalescence, central control, and co-ordination.

That this beneficent process has begun, if only begun, is sufficiently apparent from the recognition and grants which are now obtainable, as mentioned above, from the Board of Education and the Local Government Board. With the degree of control which such grants necessitate, and the limits of voluntary and State effort, we are not here concerned, except in so far as an absence of practical definition affects the status and training of the *personnel*, of which the health visitor, by whatever new name she may be called, is the type. Working under some medical supervision in any case, the hospital almoner, the medical officer of health, the lay worker herself, and the school or district nurse, have each his or her own particular views on the nature of the training which is advisable. If we examine, quite briefly, these points of view, a general principle seems to emerge which may prove of material value.

It will be immediately noted that for the type of work now required for what may be generally described as "mothercraft and child welfare" schemes, two elements of knowledge are required. The one is training of a nursing, or semi-medical character; the other is training in social work, in the sense in which that term is understood by, for instance, Miss Cummins, the lady almoner at St. Thomas's Hospital, or by the Organising Secretary of the Charity Organisation Society. If, then, we examine the divergent points of view of the various officers mentioned in the preceding paragraph, we shall see that they divide themselves broadly into two divisions, the one represented by the medical and nursing officers, and the other represented by the social workers or almoners. Which view is the more correct—or, to put it in the form of a question, are those correct who say that a health visitor must have a nurse's professional training, or are those right who contend that a trained nurse is too professional to be of the best value in dealing with the social facts that are of such importance in work among necessarily poor women, who, largely in their own homes, form the material with which the health visitor deals?

It is only necessary to state the divergent views thus simply to see that each is really not the opposite, but the complement of the other. We therefore take the training of the health visitor as a typical point to show that, until all such centres, no matter what they choose to call themselves, are properly centralised, either by affiliation to the hospitals of their district or under the control of the local medical officer of health, the existing discrepancies of standard, and more or less haphazard methods of administration and training, will not permanently be checked.

